# Auto-analysis system for graphite morphology of grey cast iron

**DOI:** 10.1155/S1463924603000154

**Published:** 2003

**Authors:** Hong Jiang, Yiyong Tan, Junfeng Lei, Libo Zeng, Zelan Zhang, Jiming Hu

**Affiliations:** 1 Department of Analysis-Measurement Science Wuhan University Wuhan 430072 China; 2 Department of Electronic Engineering Wuhan University Wuhan 430072 China

## Abstract

The current method to classify graphite morphology types of grey cast iron is based on traditional subjective observation, and it cannot be used for quantitative analysis. Since microstructures have a great effect on the mechanical properties of grey cast iron and different types have totally different characters, six types of grey cast iron are discussed and an image-processing software subsystem that performs the classification and quantitative analysis automatically based on a kind of composed feature vector and artificial neural network (ANN) is described. There are three kinds of texture features: fractal dimension, roughness and two-dimension autoregression, which are used as an extracted feature input vector of ANN classifier. Compared with using only one, the checkout correct precision increased greatly. On the other hand, to achieve the quantitative analysis and show the different types clearly, the region segmentation idea was applied to the system. The percentages of the regions with different type are reported correctly. Furthermore, this paper tentatively introduces a new empirical method to decide the number of ANN hidden nodes, which are usually considered as a difficulty in ANN structure decision. It was found that the optimum hidden node number of the experimental data was the same as that obtained using the new method.


Journal of Automated Methods & Management in Chemistry
Vol. 25, No. 4 (July–August 2003) pp. 87–92

Taylor & Francis

Taylor & Francis Group

### Auto-analysis system for graphite morphology of grey cast iron

**Hong Jiang[1], Yiyong Tan[2], Junfeng Lei[2], Libo Zeng[2], Zelan Zhang[2] and Jiming Hu[1]***

*[1]Department of Analysis-Measurement Science and [2]Department of Electronic Engineering, Wuhan University, Wuhan 430072, P. R. China*

*The current method to classify graphite morphology types of grey cast iron is based on traditional subjective observation, and it cannot be used for quantitative analysis. Since microstructures have a great effect on the mechanical properties of grey cast iron and different types have totally different characters, six types of grey cast iron are discussed and an image-processing software subsystem that performs the classification and quantitative analysis automatically based on a kind of composed feature vector and artificial neural network (ANN) is described. There are three kinds of texture features: fractal dimension, roughness and two-dimension autoregression, which are used as an extracted feature input vector of ANN classifier. Compared with using only one, the checkout correct precision increased greatly. On the other hand, to achieve the quantitative analysis and show the different types clearly, the region segmentation idea was applied to the system. The percentages of the regions with different type are reported correctly. Furthermore, this paper tentatively introduces a new empirical method to decide the number of ANN hidden nodes, which are usually considered as a difficulty in ANN structure decision. It was found that the optimum hidden node number of the experimental data was the same as that obtained using the new method.*

### Introduction

Grey cast iron has always played an important role both in materials science and metallurgy. Much research is interested in it because of its popular use in industry [1]. With the development of theoretical research, computer technologies are much more widely used in metallurgy than before, but they are still limited. Most of the applications focus on data simulation, especially in different types of detailed industrial processes. Some applications also focus on design and management, and a few focus on the forecast and analysis of properties [2,3].

The present research supplies a good example in the field of automatic properties analysis in the iron and steel industry. First, it is important to obtain information on the microstructure and microcomponents of iron and steel and classify them automatically so that different treatments can be applied to them easily. Most properties of iron or steel are determined by their microstructures and microcomponents. Second, some general methods and image analysis instruments are summarized in [4]. However, the authors focus just on the necessity of quantitative grade assessment. Next, in [4], most methods mentioned were designed through a subjective comparison with the standard images. Nevertheless, the automatic or semi-automatic image analysis instruments only had very limited functions. On the other hand, artificial intelligence techniques, especially the neural network and expert system, as a tool are becoming increasingly more popular and important [5]. It is a kind of non-linear technique that began to develop quickly in the mid-1980s and which has produced satisfactory results in many fields. For example, there are several applications of an artificial neural network (ANN) in quality prediction of grey cast iron [6,7], but there is no report on graphite morphology analysis of grey cast iron.

Above all, this paper describes an automatic image-processing and analysis software subsystem that supplies a good tool for auto-analysis, takes the ANN into the analysis and performs the auto-analysis of grey cast iron. This subsystem is a part of the metallurgic analytical system that completes metallurgical structure recognition, quantitative grade assessment on non-metallic inclusion in iron and steel, and also some other analytical functions.

### Graphite morphology type standard and requirement

### *Graphite morphology*

For grey cast iron, the graphite distribution takes on the appearance of a sheet. According to ISO 945-1975, the graphite morphology of grey cast iron can be divided into six types:

- Type A: takes on an equably distributed sheet-like appearance. This is typical.
- Type B: chrysanthemum-like and uneven.
- Type C: nubbly.
- Type D: can be ramiform, and punctuated or small sheet-like between the ramifications.
- Type E: can be ramiform, and large sheet-like between the ramifications, uneven but showing direction.
- Type F: asteroid.

The differences in graphite morphology result from the changes in chemical components and cooling conditions. At the same time, such microstructures have a great effect on the mechanical properties of grey cast iron. For example, type A has the best mechanical properties among the six types. Much research has been done

* To whom correspondence should be addressed.
e-mail: jmhu@whu.edu.cn on the properties and treatment methods of different graphite-morphology types [8]. Grey cast iron with different graphite morphology types can be used in different ways.

*Physical requirement*

To obtain a general and standard analysis result, not all samples can be pretreated in a chemical way based on ISO 945-1975. Moreover, the enlargement factor of the microscope should be $\times 100$. All images in figure 1 are obtained from a metallographic microscope under the above conditions. Types A, B, D and E are typical structures, and the classification of the images in figure 1 were decided on subjectively according to ISO 945-1975.

### Graphite morphology auto-analysis

The main structure of the processing procedure of the system is as shown in figure 2.

The flow chart of the subsystem as shown in figure 3, which is divided into four parts: image pretreatment model, feature extraction model, neural network classifier model and result reporting model. Among them, the neural network classifier model works on a BP (back-propagation) neural network.

In figure 3, the Feature Extraction is designed as two modes: manu- and automode. Manumode is used to build an expert knowledge database. It builds a standard sample database through a training neural network based on standard samples with types already known.

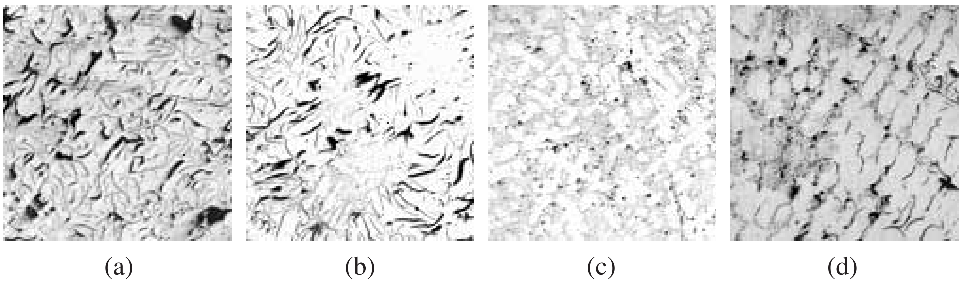

*Figure 1. Original grey value images of graphite morphology. Types (a) A, (b) B, (c) D, (d) E.*

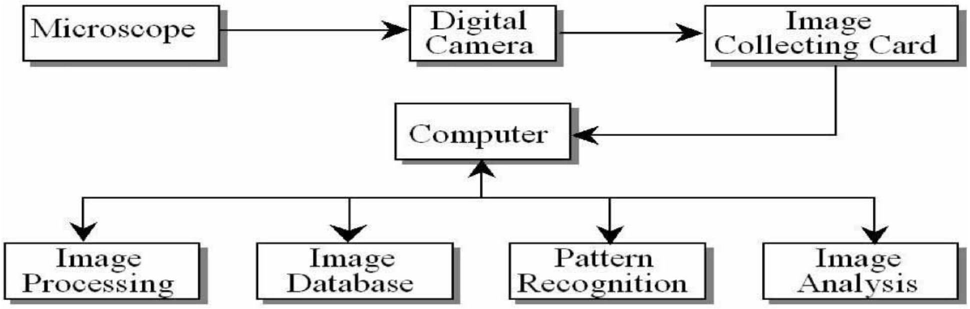

*Figure 2. Structure of the processing procedure.*

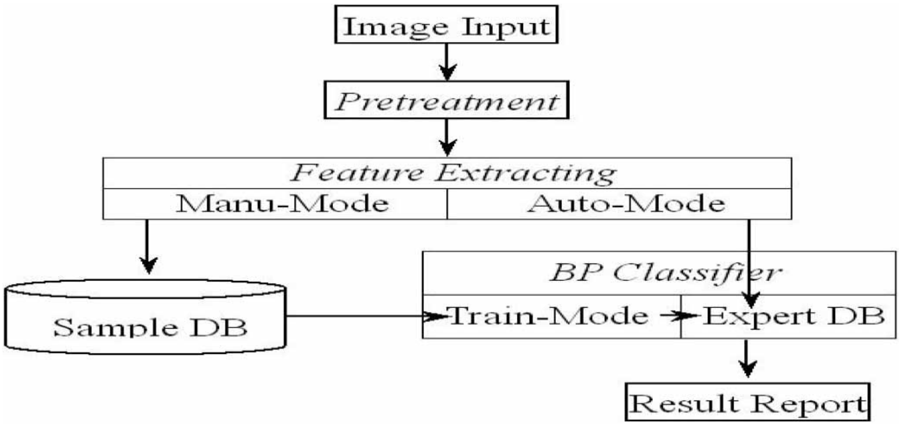

*Figure 3. Auto-analysis system flow chart.*

Automode is used for auto-analysis according to the standard sample database built up by manumode.

*Pretreatment*

Three factors must be taken into account when the auto-analysis system is used to classify the graphite morphology types of grey cast iron. First, unavoidably, there are existing kinds of noise, various photometric anomalies and bad image quality resulting from other factors. Second, as figure 1 shows, the grey value between different images varies greatly, as does that within the same image. Such unevenness makes auto-analysis hard to achieve and makes the result imprecise. Third, not all information in the image is useful in recognition, and not all useful information is easy to get. All these make pretreatment a prerequisite in the whole analysis process.

Thus, we need to reduce noise, improve image quality, make the images even, reduce the useless information and, at the same time, enhance the useful information.

Pretreatment includes: binarization, edge enhancing, unevenness sharpening and background correcting. The result of pretreatment on the images in figure 1 is shown in figure 4.

*Feature extracting*

Feature extracting is the key to good recognition. Because the graphite morphology of grey cast iron has strong textural characteristics, the work here is based on texture analysis. On the other hand, there are many methods used for texture analysis and most do not give clear-cut results. Therefore, here, we chose from the available methods according to the reference documents and tested them on our testing platform. Finally, three methods are selected and combined to form a features vector. Compared with using only one feature, this combined features vector shows a better result and greatly increase the precision of a correct recognition.

The three kinds of textural features are fractal dimension, roughness and two-dimension autoregression.

*Fractal dimension.* In [9], some research on the fractal characteristic of cast iron is introduced. According to this research, a fractal dimension is a valid descriptor for grey cast iron analysis. The fractal dimension got from the Box-Counting method is described [10]:

$$D_B^F = \lim_{\delta \to 0} \frac{\log N_\delta(F)}{-\log \delta}, \qquad (1)$$

where $N_\delta(F)$ is the minimum number of the set with diameter $d$, i.e. the set is the minimum one that can overlay the image or given processing window $F$.

*Roughness.* Roughness of texture is got from a correlation of the image [11].

We set the analysis window size as an $11 \times 11$ pixel. That is, the pixel is defined in the window $(x, y)$ and the centre pixel is (0, 0), then $x, y = 0, +1, -1, +2, -2, +3, -3, +4, -4, +5, -5$, compared with the centre pixel ($x, y$ show the direction of the horizon and the vertical):

$$R_{(i,j)} = \sum_{x=-5}^{5} \sum_{y=-5}^{5} x^2 y^2 A_{(i,j)}^{(x,y)}, \qquad (2)$$

where $A$ is the correlation coefficient of the image:

$$A_{(i,j)}^{(x,y)} = \frac{\sum_{m=i-5}^{i+5} \sum_{n=j-5}^{j+5} f(m,n) f(m-x, n-y)}{\sum_{m=i-5}^{i+5} \sum_{n=j-5}^{j+5} f^2(m,n)}, \qquad (3)$$

where $f()$ is the grey value of each pixel in the image.

*Two-dimension autoregression.* This model is described in [12]:

$$\begin{aligned} f_{(i,j)} = {} & a_{ij0} + a_{ij1} f_{(i-1,j-1)} + a_{ij2} f_{(i-1,j)} \\ & + a_{ij3} f_{(i-1,j+1)} + a_{ij4} f_{(i,j-1)} + a_{ij5} f_{(i,j+1)} \\ & + a_{ij6} f_{(i+1,j-1)} + a_{ij7} f_{(i+1,j)} + a_{ij8} f_{(i+1,j+1)}. \qquad (4) \end{aligned}$$

After extracting texture, we get a feature table or matrix M. In ANN training, the matrix M is transformed to a new matrix G described as follows:

$$G_{(i,j)} = \frac{M_{Max(j)} - M(i,j)}{M_{Max(j)} - M_{Min(j)}}, \qquad (5)$$

where $i$ and $j$ are the row and column of the feature matrix; M $(i, j)$ is the element with row $i$ and column $j$; M Max $(j)$ is the maximum element in column $j$; and M Min $(i)$ is the minimum element in row $i$.

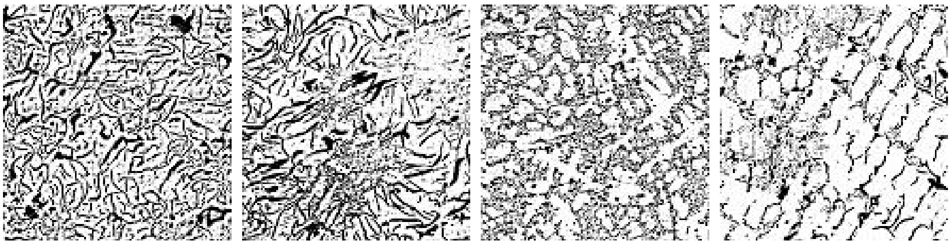

(a) (b) (c) (d)

*Figure 4. Binary texture images of graphite morphology. Types (a) A, (b) B, (c) D, (d) E.*

*Classification*

The Naïve Bays classifier method has a rigid basis in statistical theory and a very high precision of recognition, but it needs to set a priority probability of extracted features for different grey cast iron. To do this, one should have enough sample test data available and carry out much statistical work. It is time-consuming and in some applications unnecessary. In addition, if the k-nn classifier is to be used, a proper weight of the extracted features needs to be set first. Then, we have a similar task to do as in the Naïve Bays classifier. Since we only need to classify six types of grey cast iron in our application system and we want to implement a flexible auto-analysis system with high fault-tolerance ability and relatively little expert knowledge, the ANN classifier is chosen for our classification system.

The flow chart of the BP Classifier as shown in figure 5.

The classification is achieved by a kind of feed-forward ANN, which learns samples and trains itself by a BP algorithm: the error back propagation algorithm. The original model of a feed-forward multilayer back-propagation neural network is described in [13].

The BP model used here includes one hidden layer. The transfer function chosen is the tan-sigmoid transfer function.

*Hidden node*. Concerning the number of nodes in the hidden layer, there is no mature method currently in use, and in different cases different methods are used to determine the exact number of hidden nodes. How to determine hidden node numbers is always a difficulty when building a neural network structure. If the number is too small, the network may not be trained well. Conversely, if the number is too large, then the network will be slower.

Thus, a formula is invented here to determine the hidden node number. This method is based on a thorough study of our case and large testing data. It is also valuable to other similar cases. The number of hidden node is described as:

$$\mathcal{N}_H = \frac{\mathcal{N}_I + (\mathcal{N}_O, \mathcal{N}_C)_{\max}}{2}, \qquad (6)$$

where $\mathcal{N}_I$, $\mathcal{N}_O$ and $\mathcal{N}_C$ are input node number, output node number and goal class number.

The qualities of a good hidden node number are as follows:

- Big enough to keep information about goal classes. We can consider the hidden nodes as a kind of passage for information flowing from input nodes to output nodes. If the hidden nodes are too few, then the information getting to the output nodes is not enough to get a correct classification. That is, too much information is lost.
- As small as possible to keep the network running fast. On this point, not much information will be lost. It is something like using a big bottle to hold only a little water. It takes up too much space, and makes the network structure complex. A lot of time is used to calculate useless information.

Thus, we estimate that the hidden node number between the input node number and output node number or a goal-class number will be suitable. Generally, the maximum number of output nodes and the goal class number are a kind of measure of the information quantity flowing to the output nodes. In some other case, if samples for training are much more than the input node and output node numbers, then the sample number could also be considered, and the hidden node number might be increased a little, but not much. To simplify the structure, we consider only output node number, output node number and goal-class number. A test is made on the relationship of the hidden node number with a training step for a different input node number, output number and goal-class number. The training step used here is used as a measure of speed. Test results show that our method achieved great success. The testing result is shown in figure 6.

Number 1 is a hidden node number versus training step given in input node number 2, output node number 1 and goal-class number 2; number 2 is the one given such items as 5, 5 and 5; number 3 is the one given such items as 11, 3 and 6. Every training step here is the mean of training every 20 times.

Results show that the training step changes with an increase of the hide node number when given the input node number, output node number and goal-class number. The trend is reduce–increase–reduce. Obviously, the best hide node number should be the first lowest point

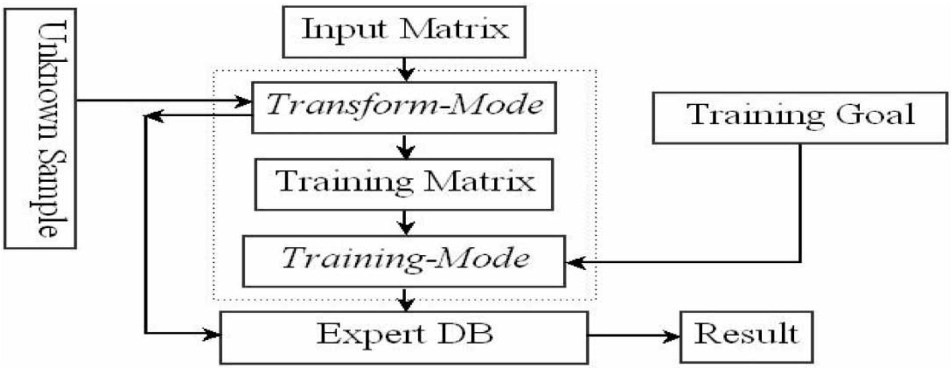

*Figure 5. Flow chart of the BP classifier.*

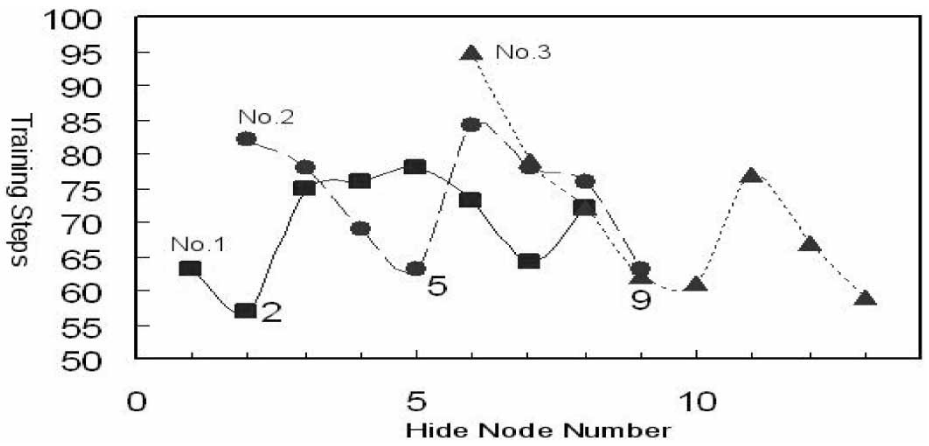

*Figure 6. ANN node number testing result.*

according to the above analysis. When it is the first lowest point, the training is fast and the node number is small.

Although this formula is an empirical formula, for the ANN is used to classify a relatively small type set, it is a very efficient and simple way to find an optimum hidden node number. We can find that the responding hidden node number of the first lowest point in the graph is exactly the same result got through formula (6). To reach higher precision, training steps will increase correspondingly, but the trend is similar and the formula can still work correctly.

*Network training algorithm.* The self-learning algorithm of the BP model is an iterative procedure as follows [14]: first, a set of weights from the network is initialized, and then a sample is input to the network and its output calculated. The difference between the calculated and expected values is used to update the weights so that the difference can be reduced. This updating process is repeated until the difference is smaller than a specified given error. After the neural network is trained by the self-learning of sufficient samples, the final weights are taken as its correct interior representation. When the final weights have been trained well, the unknown samples can then be classified.

We used the following formula to adjust our node weight of the ANN classification system:

$$\Delta W_{ij}(n+1) = \eta(\delta_i O_i) + \alpha \Delta W_{ij}(n), \qquad (7)$$

where

- $n$ iterative number,
- $\Delta W_{ij}$ weight variation between node $i$ and node $j$,
- $\eta$ learn step length,
- $\alpha$ momentum factor
- $\delta_i$ output deviation of node $i$,
- $O_i$ output value of node $i$.

The initial node weight is set by a random number. On average, after the ANN system was studied 5000 times in a training sample set of 25 ($n = 5000$, $\eta = 0.5$, $\alpha = 0.5$), a precision degree of 0.001 was reached. The size of the test set was 50 and the checkout correct rate was 92%. Compared with using only one, the checkout correct precision was greatly increased. The degree of precision was good enough for our application requirement and a higher precision depended on the collection of expert knowledge and the enlargement of the training set, in other words, network training. This problem can be overcome by collecting many samples and training the samples to build a standard recognition database.

### Applications

To achieve the quantitative analysis and show the different types clearly, the region segmentation idea [15] was applied to the system. Six different colours were used to represent different types of grey cast iron and the region with different types was painted with different colours. The percentages of the regions with different types are reported. After our auto-analysis system processed the image of a sample, the result was visualized and we could obtain information directly about how different types of grey cast iron were distributed in the sample and what percentage of each type of grey cast iron was in the sample. In fact, the precision of the classification result and the quantitative ability was good enough to undertake auto-analysis and had much advantage over the traditional manual method. This system has been tentatively used in some companies. The testing result is as shown in figure 7.

### Conclusion

Automatic analysis is an important topic in material research, and the development of computer technology provides a good tool for research in this field. This paper describes a software system that applies ANN technology to the analysis of grey cast iron and achieves quantitative analysis. Furthermore, the degree of precision depends on the collection of expert knowledge, in another words, network training. This problem can be overcome by collecting many samples and training the samples to build a standard recognition database.

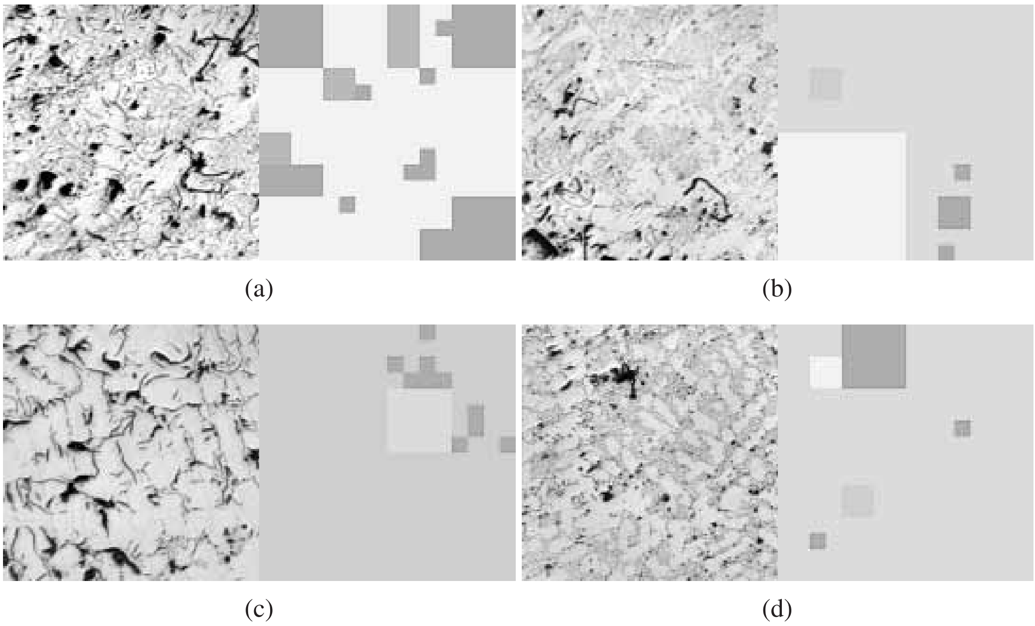

*Figure 7. Unknown sample image and the testing result comparison.*

Thus, this collection is also an essential step in putting the approach into actual application. As a side observation, the segmentation regions were not so smooth, although the arithmetic was fast. Therefore, further work will focus on this area

In conclusion, we offer a valuable system that can be applied after inputting enough expert knowledge. The quantitative analysis results are good enough, even without enough expert knowledge in the testing system. This approach is potentially a powerful and straightforward application for ANN to classify the graphite morphology types of grey cast iron. To achieve improvement and facilitate the perspective application in industry, future work will concentrate on building an expert knowledge database and on optimizing the design of the arithmetic and the software.

### Acknowledgements

Work was supported financially by the Ministry of Science and Technology, P. R. China.

### References

1. Dippenaar, R. J., *Journal of the South African Institute of Mining and Metallurgy*, **5** (1996), 91.
2. Naiyu Huang, *Casting*, **12** (1998), 3.
3. Daben Zeng, *Modern Cast Iron*, **1** (1999), 5.
4. Jixiong Liu, Meiqiang Wang and Tao Pen, *Research on Iron and Steel*, **4** (1996), 33.
5. Jiaqiang Yan, Peixin Zhang, Songgao Shan and Weiqiong Wu, *Materials Guiding Report*, **13** (1999), 15.
6. Youming Wang and Guoxiong Sun, *Material Science and Engineering*, **16** (1998), 26.
7. Li, D. Y., Liu, Y. M., Zhang, Y. T. and Meng, F. Y., *International Journal of Cast Metals Research*, **11** (1999), 391.
8. Aranzabal, J., Gutierrez, I. *et al.*, *Metallurgical and Materials Transactions* A, **28A** (1997), 1143.
9. Youming Wang and Guoxiong Sun, *Modern Cast Iron*, **1** (1998), 9.
10. Falconer, K. J., *Fractal Geometry: Mathematical Foundations and Applications* (Beijing: Dong Bei University, 1996), 58.
11. Seng Juner *et al.*, *BASIC Image Processing Program with 150 Examples* (Hefei: Chinese Science and Technology University, 1992), 278. [in Japanese]
12. Shiliang Xu, *C Common Algorithms Library* (Beijing: Tsing Hua University, 1996), 233.
13. Lippmann, R. P., *IEEE ASSP Magazine*, **April** (1987), 4.
14. Liming Zhang, *Model and Applications of Artificial Neural Networks* (Shanghai: Fudan University, 1993).
15. Baronti, S., Casina, A., Lotti, F., Favoro, L. and Roberto, V., *Computer Vision Graphics and Image Processing*, **49** (1990), 346.

